# High-Quality Draft Genome Sequence of the Multidrug-Resistant Clinical Isolate *Enterococcus faecium* VRE16

**DOI:** 10.1128/genomeA.00992-16

**Published:** 2016-09-22

**Authors:** Suelen Scarpa de Mello, Daria Van Tyne, Andrei Nicoli Gebieluca Dabul, Michael S. Gilmore, Ilana L. B. C. Camargo

**Affiliations:** a Department of Molecular Biology and Evolutionary Genetics, Federal University of São Carlos, São Carlos, São Paulo, Brazil; b Department of Microbiology and Immunobiology, Harvard Medical School, Boston, Massachusetts, USA; c Laboratory of Epidemiology and Molecular Microbiology (LEMiMo), Center for Innovation in Biodiversity and Drug Discovery, São Carlos Institute of Physics, University of São Paulo, São Carlos, São Paulo, Brazil; d Department of Ophthalmology, Harvard Medical School, Massachusetts Eye and Ear Infirmary, Boston, Massachusetts, USA

## Abstract

Specific lineages of the commensal bacterium *Enterococcus faecium* belonging to CC17, especially ST412, have been isolated from patients in several hospitals worldwide and harbor antibiotic resistance genes and virulence factors. Here, we report a high-quality draft genome sequence and highlight features of *E. faecium* VRE16, a representative of this ST.

## GENOME ANNOUNCEMENT

Infections caused by vancomycin-resistant *Enterococcus* (VRE) are increasingly difficult to treat and often cause prolonged hospital outbreaks (1). In the case of *E. faecium*, these challenges appear to be caused by the expansion and dissemination of strains belonging to the clonal complex (CC) 17 (2). In Brazil, *E. faecium* strains belonging to sequence type (ST) 412 (part of CC17) are often found in hospitalized patients (3–6). Recently, *van*A-containing *E. faecium* ST412 was found in urban rivers (Tietê River and Pinheiros River) in Sao Paulo, the largest and most populous metropolitan area in Brazil, underscoring the importance of this lineage as a major public health threat (7).

*E. faecium* VRE16 is an ST412 strain, isolated from a perianal swab collected from a male patient on 3 September 2009, during a surveillance program in the Intensive Care Unit of Risoleta Tolentino Neves Hospital–MG/Brazil. It is representative of the endemic VRE *E. faecium* strains found within this hospital in that year. VRE16 is resistant at high levels to vancomycin, teicoplanin, erythromycin, ampicillin, penicillin, and streptomycin (3).

Genomic DNA of VRE16 was extracted using a DNeasy blood and tissue kit (Qiagen, USA). DNA libraries were prepared using an Illumina Nextera XT DNA sample preparation kit (Illumina Inc., USA), with recommended modifications for 2 × 250-bp paired-end sequencing. Samples were multiplexed and sequenced on an Illumina MiSeq machine (Illumina Inc., USA). CLC Genomics Workbench version 8.0.2 (CLCBio, Denmark) was used for genome assembly, using default parameters. The genome was annotated using the NCBI Prokaryotic Genome Annotation Pipeline (8). The entire vancomycin resistance-encoding transposon Tn*1546* (10.8 kb) was amplified by long-range PCR, as described by Woodford et al. (9). Overlapping PCR was performed following the protocol of Arthur et al. (10), and the fragments were sequenced to identify the insertion sequences present in them. IS*16*, exclusively prevalent among hospital *E. faecium* strains from an international collection of isolates, was amplified by PCR using a previously described protocol (11).

Sequencing/assembly generated 8,172,175 reads, with a total size of 2,897,665 bp, an *N*_50_ value of 1,495,544 bp, a GC content of 37.8%, and 594-fold coverage.

The following antibiotic resistance genes were found by ResFinder (http://www.genomicepidemiology.org): *van*A (glycopeptide resistance), *aph(3′)-III* and *ant(6)-Ia* (aminoglycoside resistance), *msr(C)* (macrolide, lincosamide and streptogramin B resistance), and *erm(B)* (macrolide resistance). Regarding the characteristics of Tn*1546*, the element conferring vancomycin resistance, VRE16 lacks the right inverted repeat (IR_R_). IS*1251* was inserted within the *van*S-*van*H genes at the same position as already observed in other Brazilian isolates (4, 6, 7, 12), and IS*1216E* was inserted between *vanX* and *vanY* after nucleotide 8649 (position according to the reference sequence M97297.1 in NCBI). VRE16 was positive for the hospital marker IS*16* and has the virulence genes *acm* and *efaAfm.* Since CC17 represents a significant problem in hospitals worldwide, *E. faecium* VRE16 has been used as a standard strain for searching new compounds with *in vitro* antibacterial activity, as an initiative of the Center for Innovation in Biodiversity and Drug Discovery (CIBFar: http://cibfar.ifsc.usp.br/english).

### Accession number(s).

This whole-genome shotgun project has been deposited at DDBJ/ENA/GenBank under the accession number LSYW00000000. The version described in this paper is the first version, LSYW01000000.
